# Ameloblastoma: Management and Outcome

**DOI:** 10.7759/cureus.3437

**Published:** 2018-10-10

**Authors:** Mohammad Adeel, Muhammad Shaheryar Ahmed Rajput, Asif Ali Arain, Maqbool Baloch, Mumtaz Khan

**Affiliations:** 1 Head and Neck Oncology, Shaukat Khanum Memorial Cancer Hospital and Research Center, Lahore, PAK; 2 Otolaryngology, Head and Neck Surgery, Liaquat University of Medical and Health Sciences, Jamshoro, PAK; 3 Otolaryngology, Head & Neck Surgery, The Indus Hospital, Karachi, PAK; 4 Otolaryngology, Aga Khan University Hospital, Karachi, PAK

**Keywords:** orthopantomogram, free fibular flap, odontogenic tumor, ameloblastoma, opg, unicystic ameloblastoma, solid ameloblastoma, multicystic ameloblastoma, tumors and cysts of jaw

## Abstract

Introduction

Ameloblastoma is a locally destructive tumor with a propensity for recurrence if not entirely excised. Management of ameloblastoma poses a challenge for all involved in the field of head and neck surgery because successful treatment requires not only adequate resection but also a functional and aesthetically acceptable reconstruction of the residual defect.

Methods

Patients who had histologically proven ameloblastoma between 1991 and 2009 were identified from the database of Aga Khan University Hospital. A review of all medical records, radiological images, operative reports and pathology reports was undertaken.

Results

A total of 15 patients with histologically confirmed ameloblastoma were identified. Out of 15 patients nine were males and six were females with age range from 20 to 60 years (mean age 43 years). The most common symptom found in our patient group was painless facial swelling. In 13 patients the origin of tumor was mandible and in the remaining two the tumor originated from maxilla. Eleven out of 15 patients underwent segmental mandibulectomy, two had maxillectomy and two had enucleation. All patients who underwent segmental mandibulectomy required reconstruction. Reconstruction was done with microsurgical free tissue transfer in eight patients, non-vascularized iliac crest bone graft was used in one patient and two had plating only. All free flaps survived with no evidence of flap loss. The mean follow-up was eight years. There was no evidence of graft failure which was used in one patient. Complication was seen in only one of our patients in the form of plate exposure. Recurrence was seen in two of our cases who primarily underwent enucleation. All patients had satisfactory speech, cosmesis and mastication.

Conclusion

The management of ameloblastoma still poses a big challenge in spite of being the most common odontogenic tumor. In our study we have found that segmental mandibulectomy with disease-free margin of around 1 cm and immediate reconstruction with free tissue transfer have shown good results.

## Introduction

Ameloblastoma is a benign but locally aggressive tumor of epithelial origin that arises from enamel, dental follicle, periodontal ligaments or lining of odontogenic cysts [1, 2]. It is a rare head and neck tumor but it is still the most common odontogenic tumor [3].

The estimation of annual incidence of ameloblastoma is 0.5 per million population. This accounts for more or less 1% of tumors and cysts involving jaw and 10% of tumors of dental origin. Although ameloblastoma involves all age groups, peak incidence is documented in the second and sixth decade [4, 5]. The third and fourth decade is also mentioned for the peek incidence by others [6]. There is significant difference among racial groups. In Blacks more cases are seen in third decade whereas Caucasians have peak incidence during the fourth decade. The disease is most often found posteriorly in the angle of mandible and ascending ramus but can occur anywhere in the mandible or maxilla. Overall 80% of all ameloblastomas occur in the mandible and 20% in the maxilla.

The tumor is usually asymptomatic and presents itself as a slowly enlarging facial swelling. Ameloblastoma is a locally destructive tumor with a propensity for recurrence if not entirely excised. Radiological investigations are helpful in diagnosis. The orthopantomogram (OPG) is a useful first-line investigation and shows well-demarcated unilocular or multilocular expansile lucencies with a so-called ‘soap bubble’ appearance [7, 8]. Computed tomography (CT) is useful in the assessment of the extent of the tumor and cortical destruction of bone [9].

There are six histopathologic subtypes of ameloblastoma that include the follicular, plexiform, acanthomatous, granular cell, basal cell, and desmoplastic types [10, 11]. These subtypes can exist singly or in combination. The tumor is also subdivided into four variants, based on its overall histologic architecture. These include the solid, multicystic, multicystic plus solid, and unicystic types [12, 13].

Management of ameloblastoma poses a challenge for all involved in the field of head and neck surgery because successful treatment requires not only adequate resection but also a functional and aesthetically acceptable reconstruction of the residual defect. Resection with wide margins and reconstruction in the same sitting is currently accepted as the treatment of choice in most cases. Idea of conservative surgery is no longer entertained since it is associated with higher recurrence rate [14, 15].

This work was done to review a management outcome of patients of ameloblastoma who were managed at Aga Khan University Hospital, a tertiary care institute in the city of Karachi, Pakistan.

## Materials and methods

Patients who had histologically proven ameloblastoma between 1991 and 2009 were identified from the database of Aga Khan University Hospital. A review of all medical records, radiological images, operative reports and pathology reports was undertaken.

All patients had preoperative radiological investigations including OPG and CT scan of head and neck. Lower limb angiography was also performed in a few cases for those who underwent mandibular reconstruction with the free fibular flap.

In all of our cases resection of tumor was carried out followed by reconstruction. Reconstructions of the mandibular defects were achieved by free tissue transfer with the free fibular flap. The free fibular flaps were raised in the standard fashion as described in the literature. Intra-operatively, a nasogastric tube was inserted in all cases to facilitate early post-operative feeding and to avoid potential contamination of the healing oral wounds.

The multidisciplinary team involved in preoperative and post-operative care and rehabilitation included surgical, nursing, physiotherapy, dietitian and dental staff. All patients were followed up with interval imaging to assess for recurrence.

## Results

A total of 15 patients with histologically confirmed ameloblastoma were identified from data base of health information system of Aga Khan University Hospital. Out of 15 patients nine were males and six were females with age range from 20 to 60 years (mean age 43 years). The most common symptom found in our patient group was painless facial swelling. In 13 patients the origin of tumor was mandible and in the remaining two the tumor originated from maxilla. These clinical details of individual patients are shown in Table 1.

**Table 1:** Clinical presentation of all patients.

Patient	Age (Years)	Sex	Symptom	Site
1	32	M	Facial swelling	Mandible
2	46	F	Facial swelling	Mandible
3	56	M	Facial swelling	Mandible
4	55	M	Facial swelling	Mandible
5	21	F	Intraoral swelling	Mandible
6	20	M	Facial swelling and trismus	Mandible
7	60	M	Facial swelling	Maxilla
8	55	M	Facial swelling	Mandible
9	45	F	Facial swelling	Maxilla
10	51	F	Facial swelling	Mandible
11	31	M	Facial swelling	Mandible
12	45	F	Facial swelling	Mandible
13	46	F	Facial swelling	Mandible
14	38	M	Facial and intraoral swelling	Mandible
15	49	M	Intraoral swelling	Mandible

Eleven out of 15 patients underwent segmental mandibulectomy, two had maxillectomy and two had enucleation. All patients who underwent segmental mandibulectomy required reconstruction. Reconstruction was done with microsurgical free tissue transfer in eight patients, non-vascularized iliac crest bone graft was used in one patient and two had AO plating without free tissue transfer. All free flaps survived with no evidence of flap loss. The mean follow-up was eight years. There was no evidence of graft failure; the iliac crest bone graft was used in only one patient. Complication was seen in only one of the two patients who had reconstruction with AO plating without free tissue transfer. Recurrence was seen in two of our cases within one year of follow-up who primarily underwent enucleation; they were later operated with wide resection and AO plating. Surgical detail and outcome of individual patients are shown in Table 2.

**Table 2:** Surgical details and outcome.

Patient	Surgery	Reconstruction	Complications	Follow Up (Years)	Recurrence
1	Segmental mandibulectomy	Free fibula flap	No	5	No
2	Segmental mandibulectomy	Free fibula flap	No	6	No
3	Segmental mandibulectomy	Free fibula flap	No	8	No
4	Segmental mandibulectomy	Plating	No	11	No
5	Segmental mandibulectomy	Free fibula flap	No	4	No
6	Segmental mandibulectomy	Free fibula flap	No	9	No
7	Maxillectomy	No reconstruction	No	10	No
8	Segmental mandibulectomy	Iliac crest grafting	No	9	No
9	Maxillectomy	No reconstruction	No	6	No
10	Enucleation	No reconstruction	No	8	Yes
11	Segmental mandibulectomy	Free fibula flap	No	5	No
12	Segmental mandibulectomy	Plating	Plate exposure	11	No
13	Segmental mandibulectomy	Free fibula flap	No	6	No
14	Segmental mandibulectomy	Free fibula flap	No	7	No
15	Enucleation	No reconstruction	No	9	Yes

The average total operative time for patients requiring reconstruction by free tissue transfer was nine hours and 30 minutes. The average total operative time for patients reconstructed with bone graft or plating was three hours and 45 minutes. All patients had satisfactory speech, cosmesis and mastication.

## Discussion

Ameloblastoma is a benign but locally invasive tumor with high rate of recurrence if not resected adequately. They rarely show metastasis. There are available case reports listing metastatic ameloblastoma and ameloblastic carcinoma [16, 17]. Metastatic ameloblastoma refers to a lesion which metastasizes but the histology of both primary and metastatic tissues are benign. However, ameloblastic carcinoma on the other hand has histological features of a carcinoma.

There are various methods of treatment of ameloblastoma which are broadly divided into two types that include a conservative approach such as enucleation and a radical approach with wide local excision and reconstruction. Recurrence is well known complication associated with inadequate treatment of ameloblastoma [18]. Considering lesser aggressiveness of this tumor, enucleation had been reported as adequate treatment for unicystic type of lesions and recurrence rate had been reported low [19]. However, it should be noted that a variant of unicystic ameloblastoma in which there is mural infiltration by epithelial cells is associated with higher recurrence rate and needs wide excision of lesion for adequate treatment [20, 21].

**Figure 1:**
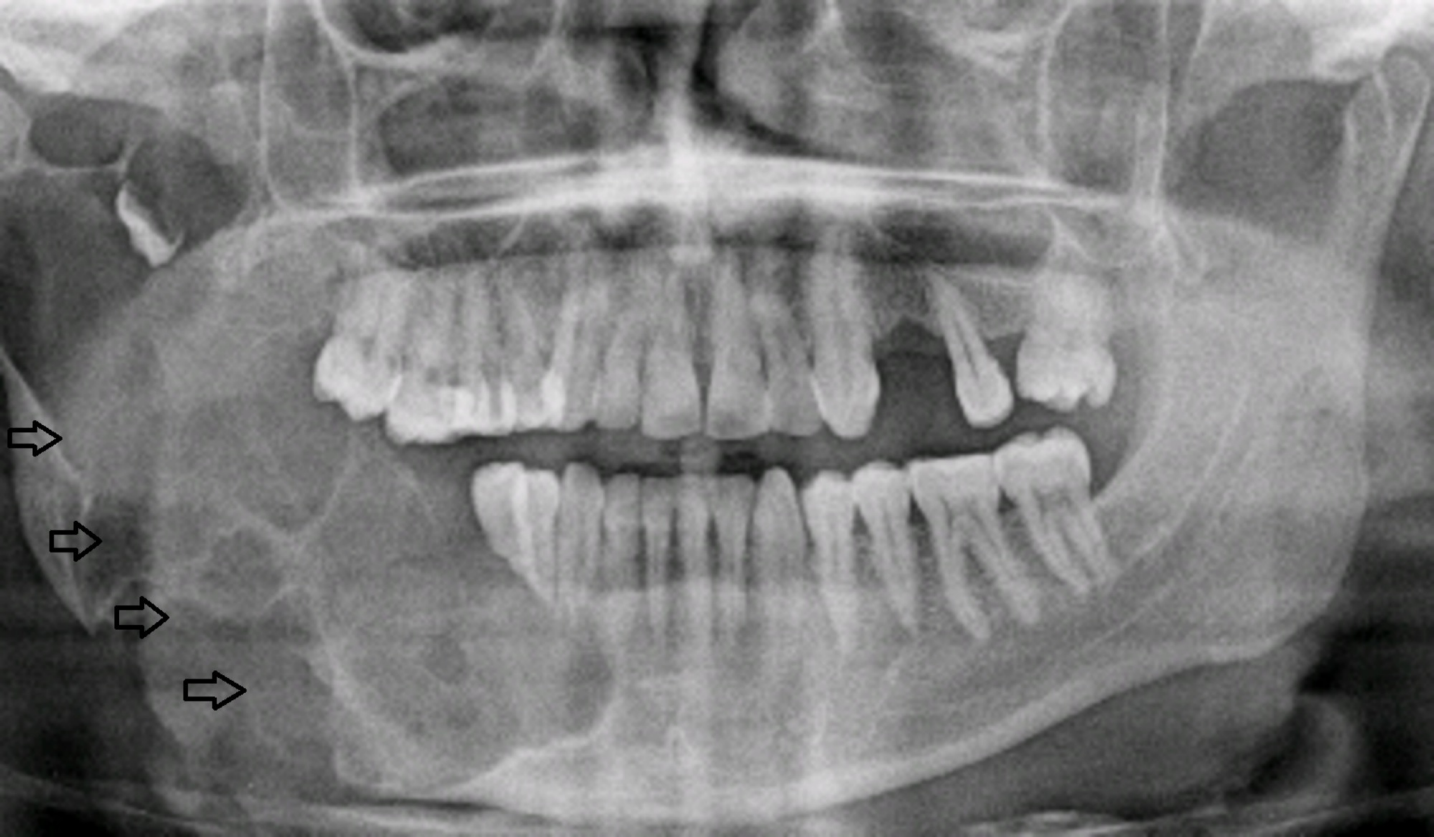
Patient 14. A 38-year-old male with right-sided facial swelling. Preoperative orthopantomogram revealed multilocular lucencies (arrows) on the right side.

In contrast to unicystic variant, multicystic ameloblastomas have shown high incidence of recurrence. In literature, reported recurrence rates of such variant are considerably higher [22]. Preoperative OPG of a patient with multicystic ameloblastoma is shown in Figure 1.

**Figure 2:**
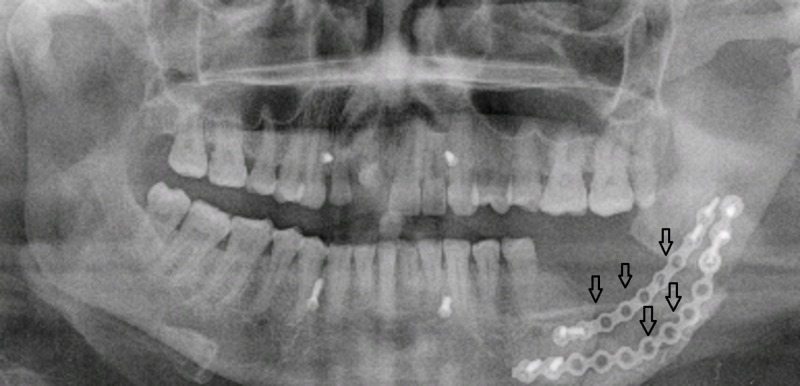
Patient 4. A 55-year-old male with left-sided facial swelling. Postoperative orthopantomogram shows reconstruction of mandible with plating (arrows).

Segmental mandibulectomy with removal of 1-2 cm disease-free bone with immediate reconstruction is considered as an ideal treatment for ameloblastoma. This gives good cosmetic results and also addresses speech and eating problems [23]. Immediate reconstruction with use of plating is shown in Figure 2.

**Figure 3:**
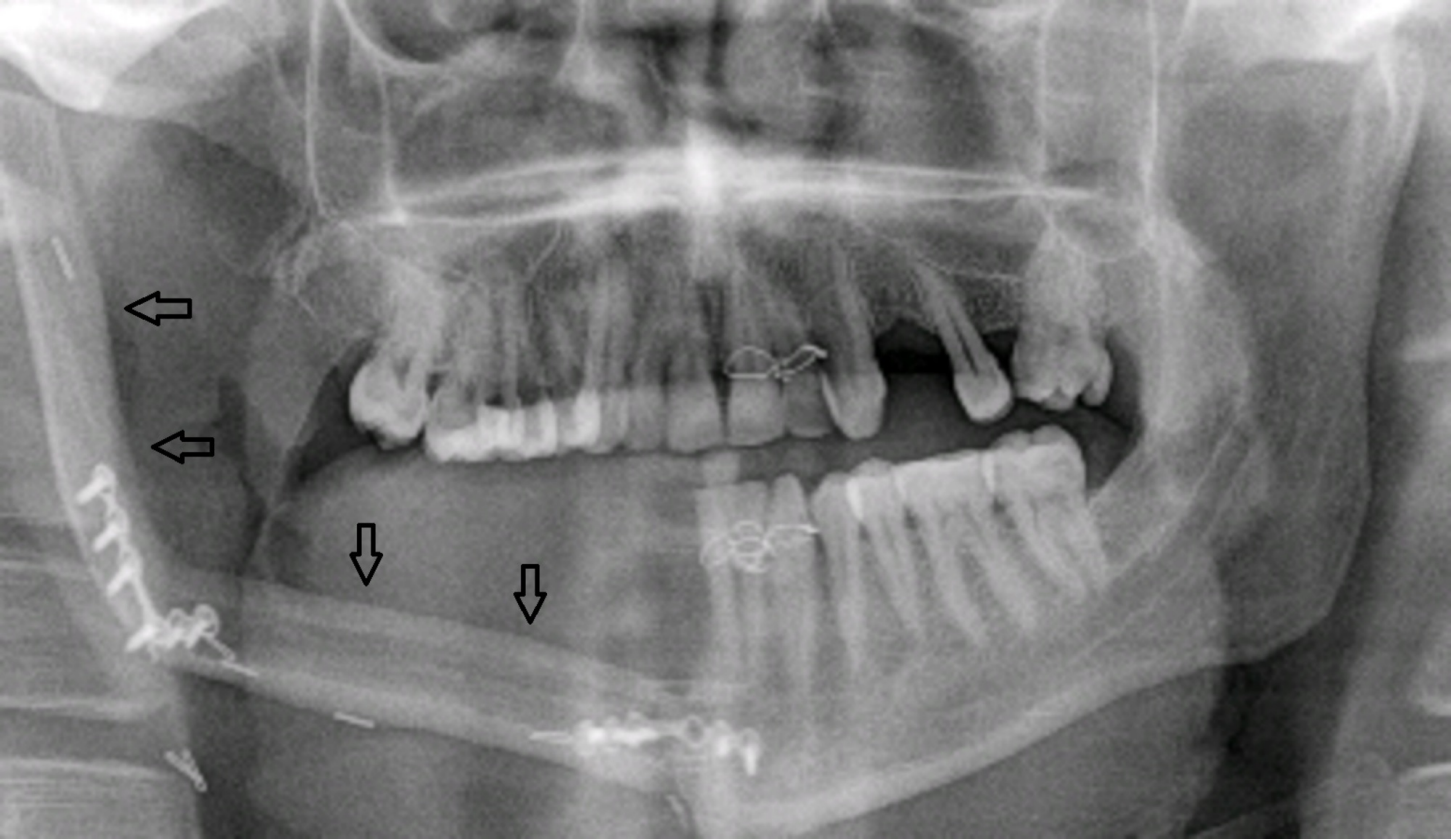
Patient 11. A 31-year-old male. Postoperative orthopantomogram shows position of free fibular flap (arrows).

The revolutions in the field of reconstructive microsurgery made free tissue transfer the method of choice for reconstruction of bony defect. In addition to covering large composite bony defects the free fibular flap also gives good aesthetic and functional outcomes with options for dental rehabilitation. Reconstruction of a mandibular defect with free fibular flap is shown in Figure 3.

## Conclusions

The management of ameloblastoma still poses a big challenge in spite of being the most common odontogenic tumor. In our study, we have found that segmental mandibulectomy with disease-free margin of around 1 cm and immediate reconstruction with free tissue transfer have shown good results.

## References

[1] Mendenhall WM, Werning JW, Fernandes R, Malyapa RS, Mendenhall NP (2007). Ameloblastoma. Am J Clin Oncol.

[2] Thompson LD (2003). Ameloblastoma. Ear Nose Throat J.

[3] Johnson NR, Gannon OM, Savage NW, Batstone MD (2014). Frequency of odontogenic cysts and tumors: a systematic review. J Investig Clin Dent.

[4] Oomens MA, van der Waal I (2014). Epidemiology of ameloblastomas of the jaws; a report from the Netherlands. Med Oral Patol Oral Cir Bucal.

[5] Gerzenshtein J, Zhang F, Caplan J, Anand V, Lineaweaver W (2006). Immediate mandibular reconstruction with microsurgical fibula flap transfer following wide resection for ameloblastoma. J Craniofac Surg.

[6] Adekeye EO, Lavery KM (1986). Recurrent ameloblastoma of the maxillo-facial region: clinical features and treatment. J Maxillofac Surg.

[7] Ruquaya M, Singh VP (2014). Ameloblastoma—a locally destructive and invasive tumour—review of literature. Int J Otolaryngol Head Neck Surg.

[8] Underhill TE, Katz JO, Pope TL, Dunlap CL (1992). Radiologic findings of diseases involving the maxilla and mandible. AJR Am J Roentgenol.

[9] Drevelengas A, Eleftheriadis J, Kalaitzoglou I, Palladas P, Lazaridis N (1994). Imaging of maxillomandibular ameloblastoma. Eur Radiol.

[10] Eversole LR, Leider AS, Hansen LS (1984). Ameloblastomas with pronounced desmoplasia. J Oral Maxillofac Surg.

[11] Aisenberg MS (1953). Histopathology of ameloblastomas. Oral Surg Oral Med Oral Pathol.

[12] McClary AC, West RB, McClary AC (2016). Ameloblastoma: a clinical review and trends in management. Eur Arch Otorhinolaryngol.

[13] Zhang J, Gu Z, Jiang L, Zhao J, Tian M, Zhou J, Duan Y (2010). Ameloblastoma in children and adolescents. Br J Oral Maxillofac Surg.

[14] Almeida RA, Andrade ES, Barbalho JC, Vajgel A, Vasconcelos BC (2016). Recurrence rate following treatment for primary multicystic ameloblastoma: systematic review and meta-analysis. Int J Oral Maxillofac Surg.

[15] Hertog D, van der Waal I (2010). Ameloblastoma of the jaws: a critical reappraisal based on a 40-years single institution experience. Oral Oncol.

[16] Gunaratne DA, Coleman HG, Lim L, Morgan GJ (2015). Ameloblastic carcinoma. Am J Case Rep.

[17] Benlyazid A, Lacroix-Triki M, Aziza R, Gomez-Brouchet A, Guichard M, Sarini J (2007). Ameloblastic carcinoma of the maxilla: case report and review of the literature. Oral Surg Oral Med Oral Pathol Oral Radiol Endod.

[18] Tamme T, Tiigimäe J, Leibur E (2010). Mandibular ameloblastoma: a 28-years retrospective study of the surgical treatment results. Minerva Stomatol.

[19] Gardner DG, Corio RL (1984). Plexiform unicystic ameloblastoma. A variant of ameloblastoma with a low-recurrence rate after enucleation. Cancer.

[20] Chae MP, Smoll NR, Hunter-Smith DJ, Rozen WM (2015). Establishing the natural history and growth rate of ameloblastoma with implications for management: systematic review and meta-analysis. PLoS One.

[21] Li TJ, Wu YT, Yu SF, Yu GY (2000). Unicystic ameloblastoma: a clinicopathologic study of 33 Chinese patients. Am J Surg Pathol.

[22] Antonoglou GN, Sándor GK (2015). Recurrence rates of intraosseous ameloblastomas of the jaws: a systematic review of conservative versus aggressive treatment approaches and meta-analysis of non-randomized studies. J Craniomaxillofac Surg.

[23] Urken ML, Buchbinder D, Costantino PD, Sinha U, Okay D, Lawson W, Biller HF (1998). Oromandibular reconstruction using microvascular composite flaps: report of 210 cases. Arch Otolaryngol Head Neck Surg.

